# Human Dynamics Research in GIScience: challenges and opportunities

**DOI:** 10.1007/s43762-024-00144-y

**Published:** 2024-11-06

**Authors:** Shih-Lung Shaw, Xinyue Ye, Michael Goodchild, Dan Sui

**Affiliations:** 1 Department of Geography & Sustainability, University of Tennessee, Knoxville, USA; 2 Department of Landscape Architecture and Urban Planning & Center for Geospatial Sciences, Applications, and Technology, Texas A&M University, College Station, USA; 3 Department of Geography, University of California, Santa Barbara, USA; 4 Office of Senior Vice President for Research and Innovation, Virginia Tech, Blacksburg, USA

**Keywords:** Human dynamics, Hybrid physical-virtual world, Ethics, Analytical platforms

## Abstract

The Symposium on Human Dynamics Research, first organized at the 2015 AAG Meeting in Chicago, celebrated its 10th anniversary at the 2024 AAG Meeting in Honolulu, marking a decade of transformative advancements in the field. Over the past decade, the focus of human dynamics research has shifted from traditional spatial-temporal analyses to sophisticated modeling of human behavior in a hybrid physical-virtual world. This evolving field now examines the intricate interdependencies between physical and digital environments, addressing critical issues such as urban resilience, public health, social equity, and community sustainability. The symposium emphasized the growing importance of interdisciplinary collaboration, advanced data-driven analytical platforms, and innovative theoretical frameworks to better understand human interactions across these spaces. As human dynamics continue to shape global urban systems, these advancements are pivotal for future research and real-world problem-solving, offering novel insights into the interconnectedness of mobility, technology, and societal well-being in a rapidly changing world.

The Symposium on Human Dynamics Research was first organized during the 2015 AAG Meeting in Chicago, Illinois, and marked its 10th anniversary at the 2024 AAG Meeting in Honolulu, Hawaii. This milestone event provided a unique opportunity to reflect on a decade of progress in the field and to chart a bold course for future research. A special issue titled ‘Human Dynamics in a Mobile and Big Data Era,’ published by International Journal of Geographical Information Science in 2016, was one of the first major outcomes of the Symposium, featuring pioneering studies that established foundational concepts and methodologies for understanding human dynamics in the context of rapid technological changes (Shaw et al., 2016).

Over the past decade, the field of human dynamics research has matured significantly, evolving from traditional spatial-temporal analyses to incorporating the complexities introduced by smart technologies and big data (Wang et al., 2016). This evolution has integrated diverse fields such as civil engineering, computer science, geography, sociology, and urban planning (Shaw & Sui, 2018; Shaw, 2022). The growing convergence of the physical and the virtual realms, driven by advances in artificial intelligence, machine learning, and data science, has transformed our understanding of human behavior and interactions (Wu et al., 2023). In this hybrid physical-virtual world, human activities are no longer confined to physical spaces but extend into virtual environments that shape and are shaped by human behavior, creating intricate feedback loops that influence everything from mobility patterns to social interactions and community dynamics from local to global level (Li et al., 2017; Wu et al., 2024).

Human dynamics research today is not just about mapping movements or interactions; it is about understanding the complex interdependencies between physical and virtual spaces. This holistic perspective is crucial for addressing contemporary challenges, such as urban resilience, public health, and social equity, which require a nuanced understanding of how people experience and interact with each other and their environments. For example, the growing maturity of smart technologies has transformed human mobility, influencing how people navigate and experience both urban and virtual spaces. These changes demand new conceptual frameworks beyond traditional absolute space and physical place.

Conducting human dynamics research presents many challenges, including the need for interdisciplinary collaboration, the development of new data collection and analysis methods, and the creation of theoretical approaches that can accommodate the complexity of hybrid spaces. In the meantime, these challenges also represent opportunities for innovation and further growth of human dynamics research. By engaging with these challenges, we can push the boundaries of what is possible in this field and develop new insights that will have far-reaching implications for research and practice. Below is a list of some key challenges and associated opportunities that can guide future research in human dynamics. These highlights emphasize the need to embrace interdisciplinary collaboration and methodological innovation to address the evolving complexities of human dynamics in a hybrid world.

1. **Human needs as the foundation.**

Challenges: Humans must fulfill various needs in their lives. Maslow (1958) suggests a hierarchy of human needs ranging from physiological needs (e.g., food, water, sleep), safety needs (e.g., employment, health, property), love/belonging needs (e.g., family, friends), esteem needs (e.g., achievement, respect by others), to self-actualization needs (e.g., morality, creativity, problem solving). Human dynamics is the means of fulfilling these different needs. The foundation of human dynamics research therefore must be based on human needs, wants, and constraints.

Opportunities: Beyond studying the “spatiotemporal patterns” of human dynamics, we need to understand why people organize their activities in certain ways. For example, finding how activity patterns changed during a pandemic (e.g., COVID-19) is interesting; however, it would be more useful to better understand how people overcame (or failed to overcome) constraints under various mitigation strategies to fulfill their needs during a pandemic. Such an approach can help develop effective mitigation strategies while ensuring the basic needs of various population groups are met, which makes human dynamics research truly human-centered.

2. **Hybrid physical-virtual world and city representation.**

Challenges: We now live in an increasingly hybrid physical-virtual world. As we use transportation to move among different locations in physical space to fulfill some of our needs, we also use information and communication technology (ICT) to navigate among different places in virtual space to achieve other needs. One key challenge is to better understand the mutual interactions between human activities in physical space and human activities in virtual space. Recent decades have seen significant progress in creating digital city models. Today we can build detailed three-dimensional representations of structures down to spatial resolutions of centimeters and can generate photorealistic visualizations that are often indistinguishable from actual scenes. Architects have developed software to populate such representations with street trees of different, recognizable species. But largely missing from all this progress are the humans (Fotheringham, 2023): the vehicles that they use to move themselves and their goods through the city, the walkers who populate the sidewalks and crosswalks, the people who occupy the buildings, and human activities in virtual space.

Opportunities: Human dynamics research must go beyond the conventional GIS (geographic information systems) focus on “locations in physical space”. GIScience therefore should be able to handle not only human dynamics in physical space and in virtual space but also the interactions between physical space and virtual space. For example, how do we represent someone who wears an Apple Vision Pro device that engages the physical world and virtual world simultaneously? In a hybrid physical-virtual world, locations in physical space may or may not be directly relevant to activities in virtual space. For example, if people smile when they watch a video on their smartphone during a bus ride, it often does not suggest they love to ride buses. Detailed data from smartphones and sensor networks can be leveraged to enhance the realism of human behavior simulations. Improving digital city models by incorporating dynamic human activities and interactions in both physical space and virtual space can make both spaces more comprehensive and useful for various applications.

3. **Lack of theoretical frameworks.**

Challenges: How can we represent humans in current GIS software products? Do we have good approaches to dealing with humans in virtual space? Do we have proper theoretical frameworks to address humans in a hybrid physical-virtual world? GIS and GIScience have not paid sufficient attention to humans in terms of representation, analysis, and visualization of human dynamics. It is critical to develop new theoretical frameworks for human dynamics in a hybrid physical-virtual world. We also need to expand the concepts of “space” and “spatial data” in GIScience beyond their traditional focus on “location in physical space”.

Opportunities:

Shaw and Sui (2020) propose a space-place (splatial) GIScience framework that considers humans at the center and integrates location in absolute space, locale in relative space, identity in relational space, and sense of place in mental space to pursue human dynamics research. This framework allows researchers to pursue various aspects of human dynamics (e.g., movements in absolute space, local context in relative space, power relationships in relational space, feelings and perceptions in mental space), and to investigate the interactions among these different aspects of human dynamics. Additional efforts of developing theoretical frameworks for human dynamics research are needed.

4. **Lack of operational analytics platforms for human dynamics.**

Challenges: We are ill-equipped with operational analytics platforms that can integrate space, time, and humans to support human dynamics research. We need functions for data representation, data management, data analysis, and data visualization (e.g., How do we represent humans and analyze/visualize human dynamics in a hybrid physical-virtual world? ). We also need functions that go beyond describing spatiotemporal patterns to gain insights and deeper understanding of the underlying processes behind the patterns. Moreover, our existing technology for wayfinding, routing and scheduling, and other dimensions of human spatial behavior is rooted in the legacy of the past. The first digital representations of US street networks were produced in the 1970s and 1980s from topographic data at scales of 1:24000, sufficient to capture a city as a network of street centerlines with a positional accuracy comparable to that of a simple GPS receiver and the size of a car or bicycle, and thus adequate for vehicle wayfinding. But these databases missed many of the features that can be critical to specific applications: for example, the availability of parking spaces, the locations of curb cuts or traffic lights, or data on temporary congestion. Although much progress has been made, and today’s routing apps include many of these kinds of detail, there remains a large gap between the needs of individual movement and the information that can be obtained through the use of online services.

Opportunities: Efforts at developing space-time GIS based on the time-geographic approach for tracking/trajectory data have made some progress in addressing this challenge (e.g., Yu & Shaw, 2008, Shaw & Yu, 2009). However, conventional time geography focuses on physical space and has limitations in dealing with human dynamics in a hybrid physical-virtual world (Shaw, 2023). In addition, there are opportunities for developing new analytical functions based on other approaches and technologies (e.g., AI, wearable devices) to help us better understand human dynamics in today’s world (e.g., Feng et al., 2024, Zhao et al., 2024).

Recently major efforts have been made to fill the human needs, many of them under the term HDMap (Elghazaly, 2023), for high-density or high-definition mapping. These efforts rely on imagery captured by suitably equipped vehicles, together with artificial intelligence to identify features of interest. HDMap is in part motivated by the arrival of autonomous vehicles, and their need for much richer and finer-resolution data than those provided for traditional wayfinding. But it also can convey valuable benefits to users with special needs, such as the elderly, mobility-impaired individuals, or pedestrians with concern for personal safety. Fine-resolution imagery can also support research into automated property assessment and detailed urban planning. In the future, we can expect guidance apps to be much more accommodating to special needs, and to support a range of objective functions that go well beyond the current trip length and time to include predicted traffic delays, the presence of shade and resting places for pedestrians and cyclists, energy consumption, air quality, and personal safety.

5. **Data access and privacy/ethics concerns.**

Challenges: Another key challenge to the human dynamics research community is where to get data in support of our research. Although huge amounts of data have been constantly collected by private companies and governmental agencies, most researchers do not have access to much of the data. In the meantime, we face challenges of protecting locational privacy and practicing ethics while using sensitive data to conduct human dynamics research. It is unlikely that perfect solutions will be found to these challenges. Instead, we need to find effective solutions that balance data access needs and privacy/ethics concerns.

Opportunities: Some potential opportunities are available to address both the data access needs and the privacy/ethics concerns in human dynamics research. For example, one approach could be to establish privacy-preserving data centers modeled after the U.S. Census Bureau’s 53 Federal Statistical Research Data Centers, but with a stronger focus on secure, controlled access to sensitive locational and behavioral data (Wu & Li, 2024). These centers could leverage emerging technologies such as differential privacy, homomorphic encryption, and federated learning to ensure that sensitive information is protected while still enabling meaningful analysis. Such data centers could also include audit mechanisms to track data usage and ensure compliance with ethics standards, providing transparency to both data providers and the public. Another alternative is to have the research community build its own decentralized, privacy-respecting data collection and sharing platforms, similar to Northeastern University’s National Internet Observatory. These platforms could implement zero-trust architecture, where each data request must be authenticated and authorized, and could employ user-consent models that give individuals greater control over their data. Additionally, the platforms could adopt blockchain technology to create immutable records of data access and usage, ensuring that researchers adhere to ethical guidelines when handling sensitive information. Moreover, human dynamics research should not rely on big data alone but should embrace the concept of “responsible data science,” where small data (e.g., survey data, interview data) play a crucial role. Combining small data with big data can offer more robust insights while maintaining the integrity of individual privacy. For instance, small data can be collected with explicit consent and used to validate findings from big data, mitigating concerns about algorithmic bias and ensuring a more ethical application of data analytics. Additionally, researchers can employ synthetic data generation techniques to create realistic yet non-identifiable datasets for analysis, reducing the risks of exposing personal information.

6. **Digital twins for human dynamics research.**

Challenges: Digital twins often fail to include dynamic human behavior due to the complexities of simulating such activities accurately. Originating in manufacturing, the concept has been applied widely in the sciences, especially in urban informatics (Goodchild et al., 2024). The term "digital twin" suggests a perfect replication of a city's static and dynamic aspects, which is not feasible. The term digital twin is to some extent a misnomer, implying as it does the ability represent both the static (how the city looks) and dynamic (how the city works). Yet it is impossible for any digital representation to capture all aspects of a city perfectly, and for any model of process to exactly replicate the city’s operations. The streets visualized in digital twins remain essentially empty, because of the difficulties of simulating human behavior. Thus, digital twins will at best be fit only for certain purposes: those for which the uncertainties inherent in the digital twin’s predictions are too high to have meaningful impact on any conclusions or decisions. Moreover, the development of digital twins for cities will create numerous challenges, including issues of governance, ownership, and the sharing of proprietary data. Additionally, complications may arise when multiple digital twins offer predictions about the same issues in the same places and at the same times. Urban Digital Twins (UDTs) have been identified as a promising technology to achieve transformative positive urban change. However, the influence of UDT technology on community resilience and adaptation planning, particularly concerning human dynamics, remains unclear. There is a pressing need to integrate multi-agent interactions, artificial intelligence, and coupled natural–physical–social systems into UDTs to create a more human-centered framework that can enhance community infrastructure resilience (Ye et al. 2023; Ye et al. 2024).

Opportunities: Digital twins would package scientific knowledge about cities, supporting both analysis and prediction of urban dynamics through multilayered GIS and process models that represent various aspects of the city’s systems, such as traffic flow, urban growth, and residential selection. Digital twins must be enhanced with better simulation of human behavior to create more comprehensive and useful models for urban planning and management (Ligmann-Zielinska et al., 2020; An et al., 2023). By incorporating human dynamics, UDTs can drive positive and transformative urban changes. Learning from the history of global climate modeling is essential to managing the challenges posed by multiple sophisticated models and choosing between them. Establishing frameworks for governance and data sharing is crucial to optimizing the use of digital twins in urban planning and management.

## Summary and Conclusion

This paper provides an overview of the evolution of human dynamics research over the past decade, highlighting both the challenges and opportunities that lie ahead. The 10th anniversary of the Symposium on Human Dynamics Research offered a pivotal moment to reflect on the field’s progress and to chart a new course for future research. Over the past ten years, human dynamics research has expanded significantly, moving beyond traditional spatial-temporal analyses to embrace the complexities introduced by the convergence of smart technologies and big data. The integration of disciplines has transformed the field, enabling a deeper understanding of human behavior in a hybrid physical-virtual world. This new paradigm challenges researchers to explore the complex interdependencies between physical and virtual spaces, influencing mobility patterns, social interactions, and community dynamics.

The key challenges for human dynamics research include developing new theoretical frameworks that integrate physical and virtual spaces, creating operational analytics platforms, and addressing the ethical and logistical concerns of data access and privacy. The proposed space-place (splatial) framework offers a promising approach, synthesizing multiple dimensions of space and place to provide a more nuanced understanding of human dynamics in an era of smart technologies. This framework enables researchers to explore the fluidity between physical and virtual environments, offering new tools for understanding human behavior and interactions in a more comprehensive manner.

This paper identifies several key opportunities for advancing human dynamics research: prioritizing human needs by understanding the motivations and constraints behind activity patterns to create more human-centered approaches; enhancing digital city models to incorporate dynamic interactions in both physical and virtual spaces; developing comprehensive theoretical frameworks that capture the complexities of human dynamics in a hybrid world; creating new operational analytics platforms that integrate space, time, and human behavior for better mobility and safety insights; addressing data access and privacy concerns through balanced data-sharing practices; improving UDTs with accurate human behavior representations for urban planning; and refining mobility and wayfinding systems to cater to diverse needs, thereby enhancing overall human well-being.

As we look into the future, this paper emphasizes the critical needs for human dynamics research to improve human mobility in a hybrid physical-virtual world. The next decade will be dominated by efforts to simulate detailed movement patterns, predict congestion and emergency evacuations, and develop new traffic arrangements that accommodate multiple modes of movement, including autonomous vehicles. The goal is not just to optimize transportation systems but to enhance human well-being by making mobility safer, more efficient, and more equitable. Despite the advances in guidance applications, there is still much to be done to improve assisted wayfinding, particularly in terms of accessibility and usability for diverse populations. As we move forward, it is essential to foster transdisciplinary research collaboration to develop the operational platforms needed to support human dynamics research. By engaging with these challenges, human dynamics research can push the boundaries of what is possible, offering new insights and solutions to some of society’s most pressing issues. This paper calls for an interdisciplinary approach that integrates technical innovation with a deep understanding of human needs, ensuring that the advancements in human dynamics research will lead to transformative impacts on human well-being.

## Data Availability

Not applicable.
